# Paradoxes of person‐centred care: A discussion paper

**DOI:** 10.1002/nop2.520

**Published:** 2020-06-10

**Authors:** Martina Summer Meranius, Inger K. Holmström, Jakob Håkansson, Agneta Breitholtz, Farah Moniri, Sofia Skogevall, Karin Skoglund, Dara Rasoal

**Affiliations:** ^1^ School of Health, Care and Social Welfare Mälardalen University Västerås Sweden; ^2^ Department of Public Health and Caring Sciences Uppsala University Uppsala Sweden

**Keywords:** clinical practice, concept, evidence, healthcare, person‐centred care

## Abstract

**Aim:**

Previous research has mainly focused on the advantages of PCC and less on its disadvantages. Hence, there is a need to further explore the recent research regarding PCC from both sides. Therefore, the aim of this paper is to elucidate the advantages and disadvantages of PCC.

**Design:**

Discussion paper.

**Methods:**

We searched relevant literature published January 2000–March 2018 in PubMed, Medline, CHINAL, Scopus and Web of Science.

**Results:**

The results showed that PCC can contribute to* improved health and well‐being, improved mutual interaction in relationships, improved cost‐effectiveness and improved work environment*, while the disadvantages can involve *increased personal and financial costs, exclusion of certain groups, increased personal and financial costs, exclusion of staff's personhood and unfairness due to empathy*. An analysis of the existing literature on PCC showed paradoxes, which call for further investigation.

## 1 INTRODUCTION

As a way to strengthen individual participation in health care, approaches such as family‐, child‐, patient‐ and person‐centred care have been advocated in recent decades (Coyne, Holmström, & Söderbäck, 2018). One of these, person‐centred care (PCC), has been equated with high‐quality care (Ekman et al., 2011; McCormack & McCance, 2006). PCC places the person receiving care in the centre and focuses on their needs, strengths and weaknesses. It also respects the individual's context, history and family circumstances. Instead of viewing the patient as a passive target of care, PCC views the patient as an active part in the care and decision‐making (Ekman et al., 2011; Holmström & Röing, 2010; Leplege et al., 2007). While it shares many similarities with patient‐centred care, the goal of PCC is different: rather than aiming for a functional life, it has a meaningful life as its goal (Håkansson Eklund et al., 2018). However, different contexts require different types of centredness (Hughes, Bamford, & May, 2008). As noted by Leplege et al. (2007), PCC is a diverse and multidimensional concept.

Person‐centred care is advancing internationally. During the period 2000–2011, the number of published papers employing a PCC perspective increased from three to seven per month (Olsson, Ung, Swedberg, & Ekman, 2013). However, a literature review shows that the concepts of person‐centredness and patient‐centredness are blended and applied interchangeably (Håkansson Eklund et al., 2018).

However, the trend of advocating PCC as inherently good and as a quality goal to strive for is strong. Previous research has mainly focused on the advantages of PCC and less on its disadvantages. Hence, there is a need to further explore the recent research regarding PCC from both sides. Therefore, the aim of the present discussion paper is to elucidate the advantages and disadvantages of PCC.

## 2 DESIGN AND METHODS

For this discussion paper, we searched for empirical studies from a PCC perspective in PubMed, Medline, CHINAL, Scopus and Web of Science, (a) peer reviewed, (b) in English and (c) published January 2000–March 2018 using the term “person‐centred/ered care” in the title. The search was extended with a citation search and footnote chasing (Kacem & Mayr, 2017). Selected literature in this paper presents PCC studies with empirical evidence (i.e. inductively) for most of the reported advantages of PCC. However, disadvantages are additionally explored using other theoretical literature (i.e. abductively). The overview of findings is shown in Table 1.

**Table 1:** The outline of the advantages and disadvantages of person‐centred care in clinical settings

Advantages of person‐centred care	Items	**References**
Improved health and well‐being	Seeing and respecting the patient as a person Freedom of choice Self‐determination Self‐confidence and self‐esteem Improved quality of life Improved choice and decision‐making Reduced general fatigue and anxiety Improved functional performance Improved self‐efficacy Improved patients’ life satisfaction Reduced fatigue and anxiety Better functional performance observed	Alharbi, Carlström et al. (2014); Beauchamp & Childress (2009); Edvardsson, Winblad, & Sandman (2008); Edvardsson et al. (2011); Edvardsson, Petersson, et al. (2014); Edvardsson, Sandman, et al. (2014); Ekman et al. (2011, 2012); Feldthusen et al. (2016); Fletcher (2015); Fors et al. (2015); Fors et al. (2016); Fossey et al. (2014); Halpern et al. (2004); Hansson, Olsson et al. (2016); Holburn, Jacobson, et al. (2004); Li & Porock, (2014); Parley, (2001); Ricoeur, (1992); Sharp, McAllister, & Broadbent (2016); Sloane et al., ( 2004); Wigham et al., (2008); Rosher & Robinson (2005)
Improved mutual interaction in the relationships	Control given back to the person The importance of partnership Mutual understanding Teamwork Patient participation in care (strengthening empowerment) Improved patients’ social networks	Bolster & Manias, (2010); Dewing, (2004); Ekman et al., (2011); Fors et al. (2015, 2017, 2018); Gabrielsson et al. (2015); Hansson et al. (2016), Jansson et al. (2018); McCormack (2004); Ulin, Olsson, Wolf, & Ekman, (2016); Wilberforce et al. (2017); Wallström & Ekman, (2018); Wildevuur and Simonse (2015); Zoffmann et al. (2008)
Improved cost‐effectiveness	Reduced hospital costs Shorten hospital stays Improvement of discharge process Time efficacy	Ekman et al., (2012); Hansson et al. (2016); Ulin et al. (2016); Wildevuur & Simonse (2015)
Improved work environment	Improved working environment for staff Better job satisfaction Improved staff members capacity to meet the individuals’ needs Reduced stress of conscience among staff members Better psychological work climate	Barbosa, Sousa, Nolan, & Figueiredo, (2015); Brownie & Nancarrow, (2013); Edvardsson, Fetherstonhaugh, McAuliffe, Nay, & Chenco (2011); Edvardsson, Sandman, Sandman, & Borell (2014); Sjögren, Lindkvist, Sandman, Zingmark, & Edvardsson, (2014); Lehuluante et al. (2012); Vassbø et al., (2018)
Disadvantages of person‐centred care		
Increased personal and financial costs	More risk for patient falls More bone fractures among patients with dementia	Blom et al. (2016); Chenoweth et al. (2009); Coleman et al. (2003); Leeuwen et al. (2015); Makai et al. (2015); Metzelthin et al. (2015); Uittenbroek et al., (2018)
Exclusion of certain groups	Risk for patients with limited decision capacity to develop dependency Risk to patient's autonomy to be active in his/her care	O’Dwyer (2013); Britten et al. (2017).
Exclusion of the personhood of staff	Risk to undermine the personhood of the staff Risk for impaired well‐being of staff Risk for asymmetric relationship	Buber, (1971); Buber & Kaufmann, (1970); Mead & Bower (2000); Kadri et al. (2018).
Risk for compassion fatigue	Too much compassion can lead to compassion fatigue Excessive engagement in patient care	Håkansson Eklund et al., (2018); Coetzee & Klopper (2010); Hansen et al., (2018); Joison, (1992). Leplege et al., (2007)
Unfairness due to empathy	Empathy‐based care can be unfair since caregiver is driven by emotions rather than rationales or fairness. Some patients get more attention than others	Håkansson Eklund et al., (2018); Leplege et al. (2007); Batson, (2011); Batson, Klein, Highberger & Shaw (1995); Batson, Eklund, Chermok, Hoyt, & Ortiz, (2007); Krebs, (1975)

### 2.1 Ethics

Ethics approval was not required for this manuscript.

## 3 DISCUSSION

### 3.1 Advantages of PCC

Numerous studies have described the advantages of PCC, summarized as: *Improved health and well‐being, Improved mutual interaction in relationships, Improved cost‐effectiveness and Improved work environment*.

#### 3.1.1 Improved health and well‐being

The ethics of PCC entail reinforcing the person's resources and retaining their independence regarding their values, freedom of choice and self‐determination. These aspects are fundamental for increasing self‐esteem and quality of life. As a result, the person will experience physical and mental well‐being, increased self‐efficacy and empowerment.

Person‐centred care highlights the importance of seeing and respecting the patient as a *person –* a capable human being with resources and needs (Edvardsson, Winblad, & Sandman, 2008; Ricoeur, 1992). A *person* has been described as someone who has the capacity for self‐consciousness (Fletcher, 2015; Ricoeur, 1992). Furthermore, PCC involves considering the person's desires, values (Halpern, Johnson, Miranda, & Wells, 2004), family and social circumstances, as well as lifestyle. The purpose is to provide better health and well‐being for patients, which is in line with ethics goals (Beauchamp & Childress, 2009).

Other positive aspects for older persons, specifically, concern freedom of choice, self‐determination and purposeful living (Li & Porock, 2014). Previous research using both qualitative (Parley, 2001) and quantitative methods (Edvardsson, Petersson, Petersson, Sjogren, Lindkvist, & Sandman, 2014; Hansson, Carlström, Olsson, Nyman, & Koinberg, 2017; Holburn, Jacobson, Schwartz, Flory, & Vietze, 2004) indicated that PCC improves quality of life, life satisfaction, (Wigham et al., 2008) and well‐being (Sharp, McAllister, & Broadbent, 2016). Other effects include patients experiencing their lives differently and having more self‐confidence (Olsson, Hansson, & Ekman, 2016). Some patients showed to be more determined in their choice and decision‐making (Rosher & Robinson, 2005; Wigham et al., 2008), while others experienced reduced fatigue and anxiety (Feldthusen, Dean, Forsblad‐d’Elia, & Mannerkorpi, 2016). Patients expressed feelings of respect and recognition of personhood (Alharbi, Carlström, Ekman, Jarneborn, & Olsson, 2014), and better functional performance was observed (Ekman et al., 2012). Inpatients described that PCC had helped them boost their self‐efficacy in controlling their chronic disease (Fors, Taft, Ulin, & Ekman, 2016) and significant improvement to their empowerment and self‐efficacy was observed (Fors et al., 2015). In patients with dementia or depression, symptoms seemed to be reduced (Fossey et al., 2014; Li & Porock, 2014; Robertson & Rosher, 2006; Sloane et al., 2004). Finally, the PCC approach seemed to reduce medication use (Fossey et al., 2014).

#### 3.1.2 Improved mutual interaction in relationships

Person‐centred care has shown advantages in terms of improved mutual interactions. Previous studies stress the importance of interpersonal relationships in PCC for patients to thrive through equability and mutuality. A mutual relationship can lead to the development of a partnership, which includes shared decision‐making.

Caring relationships are described as essential in PCC, where interpersonal nursing takes place between patients and healthcare professionals (Gabrielsson, Sävenstedt, & Zingmark, 2015) who are authentic and present (McCormack, 2004). Consequently, the importance of partnership is emphasized (Ekman et al., 2011; Fors et al., 2015; Jansson, Fors, Ekman, & Ulin, 2018; Wallström & Ekman, 2018) and may be established even without face‐to‐face conversations and with vulnerable patient groups (Fors et al., 2018). For instance, in a literature concept synthesis Wilberforce et al. (Wilberforce et al., 2017) found that PCC encourages the caring relationship in the care of older persons. In addition, participation in PCC means that control is given back to the patient, who needs to be involved and to actively participate in their own care and treatment (Fors, Swedberg, Ulin, Wolf, & Ekman, 2017). The discharge process improved when the persons themselves were involved (Ulin, Olsson, Wolf, & Ekman, 2016) and had an opportunity to participate (Bolster & Manias, 2010). Furthermore, shared decision‐making strengthens the opportunity for persons to participate in a partnership (Dewing, 2004; Ekman et al., 2011) (Fors et al., 2017) and empowers them to solve problems (Zoffmann, Harder, & Kirkevold, 2008). PCC and treatment require a partnership approach to the patient and family members (Ekman et al., 2011) and could raise the understanding in clinical practice to create collaboration among institutions (Wallström & Ekman, 2018). PCC also helps patients improve their relationships and expand their social networks (Hansson et al., 2016; Wildevuur & Simonse, 2015).

#### 3.1.3 Improved cost‐effectiveness

Another advantage of PCC is cost‐effectiveness. One hospital goal is to discharge patients as early as possible. Therefore, cost reduction for patients’ hospital stays and efficient time use might be a preference for healthcare organizations. A scoping review by Wildevuur and Simonse (Wildevuur & Simonse, 2015) showed that PCC information and communication technology management had an impact on five chronic diseases through decreased hospitalization and increased cost‐effectiveness. Time efficiency was another reviewed indicator, where the impact appeared to be positive but less conclusive (Wildevuur & Simonse, 2015).

Hansson et al. (2016) reported that costs for patients receiving PCC were significantly lower than for those in the conventional care group. At workplaces where PCC was fully implemented, the outcomes showed that the length of hospital stay was reduced by 2.5 days (Ekman et al., 2012). Furthermore, the improvement involved the discharge process, with increased notifications to the institution of discharge and a confirmed discharge planning conference starting sooner than in the usual care group (Ulin et al., 2016).

#### 3.1.4 Improved work environment

Person‐centred care results in a better working environment for staff. Research indicates that burnout is highly associated with long‐term workload and stress of conscience. PCC seems to relieve staff's stress of conscience, as they are enabled to provide appropriate care.

An association has been found between person‐centredness and job satisfaction in elderly care (Barbosa, Sousa, Nolan, & Figueiredo, 2015; Brownie & Nancarrow, 2013; Edvardsson, Fetherstonhaugh, McAuliffe, Nay, & Chenco, 2011; Edvardsson, Sandman, Sandman, & Borell, 2014; Lehuluante, Nilsson, & Edvardsson, 2012; Sjögren, Lindkvist, Sandman, Zingmark, & Edvardsson, 2014; Vassbø et al., 2018) and with elderly care in an acute setting (Lehuluante et al., 2012). This could induce the importance of shifting from merely doing tasks and following organizational routines to providing PCC that promotes a good life for residents in elderly care (Edvardsson et al., 2011; Sjögren et al., 2014). Further, PCC has been shown to have a positive impact on nurses’ capacity to meet individual needs of residents in aged care (Brownie & Nancarrow, 2013).

Teamwork facilitates PCC and increases healthcare professionals’ instrumental and relational resources such as time, support and safety (Edvardsson, Sandman, et al., 2014; Sjögren et al., 2014). Working with PCC resulted in a significant reduction in healthcare professionals’ stress of conscience (Edvardsson, Sandman, et al., 2014). The effectiveness of working with PCC has shown a tendency to benefit care workers in terms of avoiding stress and burnout (Barbosa et al., 2015; Sjögren et al., 2014). PCC has further been associated with higher levels of supportive psychosocial unit climate and a higher proportion of staff with continuing education in dementia care (Sjögren et al., 2014).

### 3.2 Disadvantages of person‐centred care

There is less written regarding the disadvantages of PCC. A likely reason for this is that most researchers who publish in this field have a positive attitude towards PCC, with it being been stressed as ethically just and “the right thing to do” (Edvardsson, Watt, & Pearce, 2017; Ekman et al., 2011; Leplege et al., 2007; McCormack, 2004). Therefore, we had to dig deeper into the literature to find possible disadvantages. Due to this, we have used more cautious language, such as “might” and more theoretical reasoning as mentioned earlier. The possible disadvantages of PCC are as follows: *Increased personal and financial costs; Exclusion of certain groups; Exclusion of staff's personhood; Risk for compassion fatigue; and Unfairness due to empathy*.

#### 3.2.1 Increased personal and financial costs

Most of the existing literature sheds light on the positive sides of the PCC approach. However, alongside the advantages, PCC is one of the reasons for patient falls in clinical settings. Implementing PCC in practice demands careful thought, especially for persons with cognitive impairment. The obvious results of falls are the “costs” generated by injuries, fractures and pain. These costs not only include suffering on the patient's part and risks to patient safety but are also felt in economic terms through increased expenses for hospital care and rehabilitation.

The increased risk of falls has been shown by Chenoweth et al. (2009) and Coleman (2003). Chenoweth et al. (2009) performed a randomized control trial where urban residential sites were randomized to PCC, dementia‐care mapping or usual care. The proportions of residents with falls were significantly higher in the PCC and usual care groups than in the dementia‐care group. Furthermore, there were also significantly more falls in the PCC group than in usual care, which was in accordance with Coleman (2003). This was due to environmental enhancements such as plants and animals. Furthermore, increased costs but no statistically significant differences in health‐associated outcomes were shown (Metzelthin et al., 2015; Uittenbroek et al., 2018). Other studies have shown no significant differences in costs and outcome between PCC and usual care (Blom et al., 2016; Leeuwen et al., 2015; Makai et al., 2015).

#### 3.2.2 Exclusion of certain groups

There is no consensus regarding which group of patients PCC is best suited for. There is, however, a risk that it might be “too beneficial” for some but not for others. If one of the ideas is to involve those who are able to make informed decisions and actively participate in their care, there is a risk that some persons will take advantage of the situation, using their strong voice while others with weaker voices might be disadvantaged. There are also persons who do not wish to be involved in their care, regardless of the reason.

It might be difficult to operationalize and provide PCC in a consistent way; as O’Dwyer (2013) points out, PCC has been equated with a consumer‐based approach in elderly care. Investigating policy documents for Irish residential care standards, she found that PCC was portrayed as a hotel‐like service. Residents were seen as consumers with a right to autonomy and choice. This might be unsuitable for those older persons with a limited capacity to make informed decisions. Many older persons become dependent on others due to age‐related illnesses and/or impaired cognitive functions; this way of conceptualizing PCC does not account for the complexities involved in decision‐making for such older persons. Another example is found in the rehabilitation context, where the person must be an active partner in their rehabilitation. Not everyone wants, or is able, to be active in their rehabilitation (Britten et al., 2017).

#### 3.2.3 Exclusion of staff's personhood

Usually, the focus of PCC is on the patient/person and their rights and staff and their personhood has often been neglected. This might diminish the value of the staff as autonomous persons, which in turn might result in poor working conditions and high turnover rates.

There might hence be a risk that only patients/clients are considered persons, while staff are not. Kadri et al. (2018) found that many among the care staff in dementia care were not identified by their employing institutions as persons in their own right and that there was a general lack of acknowledgement of the moral work of caring. This means that there is a risk that the complex relationships of care work are reduced to a series of tasks, which challenges care workers’ self‐worth and self‐efficacy and negatively affects the delivery of PCC. It might be demoralizing to practice PCC in such an organization. At worst, the working conditions resulted in ethical dilemmas for staff. Therefore, Kadri et al. (2018) suggest that the status of the care staff as persons should be explicitly considered in quality standards and supported by employers’ policies. This is in conjunction with Buber's work on the I–thou relation (Buber, 1971; Buber & Kaufmann, 1970). His work has most commonly been used in underscoring the need to see the patient as a “thou” rather than an “it,” but as the professional relationship is mutual – a subject‐to‐subject relationship – it is important that the professional is not reduced to an “it.” This is in line with the Mead and Bower paper on patient‐centred care (Mead & Bower, 2000), stating that “the doctor as person” is one of the core aspects of patient‐centred care. Patient‐ and person‐centred care share many features and acknowledging a subject‐to‐subject relationship is one of them.

#### 3.2.4 Risk for compassion fatigue

There is a constant risk of healthcare staff being overloaded by duties and engagement in their patients. This increases the risk for compassion fatigue, characterized by a gradual lessening of compassion and exhaustion. Compassion fatigue includes feelings of hopelessness, constant stress and anxiety and sleeplessness or nightmares.

Compassionate care is included in the PCC approach, however, meaning a bond between the healthcare provider and the ill person (Håkansson Eklund et al., 2018). On the one hand, compassionate care is positive but on the other hand there is a risk.

Too much compassion may lead to compassion fatigue (Coetzee & Klopper, 2010; Hansen et al., 2018; Joison, 1992). Coetzee and Klopper (2010) identified risk factors, causes, processes and manifestations of compassion fatigue. It is “a state where the compassionate energy that is expended by nurses has surpassed their restorative processes” (Coetzee & Klopper, 2010). Risk factors for compassion fatigue include *contact with patients* and *use of self*. In PCC, genuine contact with patients and the use of self are definitional features. Although compassion is at the heart of all care, it is even more crucial in PCC. Therefore, the risk of compassion fatigue in PCC needs consideration (Leplege et al., 2007).

#### 3.2.5 Unfairness due to empathy

Empathy can be described as one of the fundamental aspects of PCC and as a positive value in care. However, there is a risk when one is “too empathetic.” Showing excessive empathy to certain persons can be unjust and unfair and is against the principles of ethics, even if it is unintentional.

Previous research has described that empathy is one of the constituents of PCC (Håkansson Eklund et al., 2018). In a meta‐synthesis, Håkansson Eklund et al. (2018) found empathy to be a core component of all the synthesized review articles. For example, Leplege et al. (2007) stated that in PCC “one should listen to the person with empathy” (p. 1557).

In social psychology, there is a massive body of research showing that empathy often causes people to help others, even altruistically (Batson, 2011). However, there is also evidence from experiments that empathy‐based helping directed towards one specific individual can be unfair to others who do not receive help. There are several reasons for this. First, empathy is a powerful source of motivation, which often makes people direct their attention towards one individual while forgetting others (Batson, 2011). For example, in two experiments Batson, Klein, Highberger, and Shaw (1995) asked participants to make a decision that affected the well‐being of other people. Before making this decision, some participants were induced to feel empathy for one of the other people. Participants who were not induced to feel empathy acted fairly, while those who were induced to feel empathy acted in a partial way to benefit the person for whom they felt empathy. Second, people tend to feel more empathy for people who are similar to themselves (Krebs, 1975). Third, people tend to feel more empathy for people they like (Batson, Eklund, Chermok, Hoyt, & Ortiz, 2007). Applying these experimental results to the context of healthcare, there seems to be a risk that PCC, due to its empathic nature, tends to unfairly favour patients who happen to be within the health provider's span of attention and are similar to the health provider and whom the health provider likes.

### 3.3 Potential paradoxes

The aim of this discussion paper was to elucidate the advantages and disadvantages of PCC in health care. We have identified some crucial points that seem to be paradoxes. These paradoxes need further consideration among staff when choosing the PCC approach. First, while a positive aspect of PCC is the increased well‐being of and respect for the patient as a person, negative aspects include unsuitability for specific groups and increased risks of falls.

**Figure 1:**
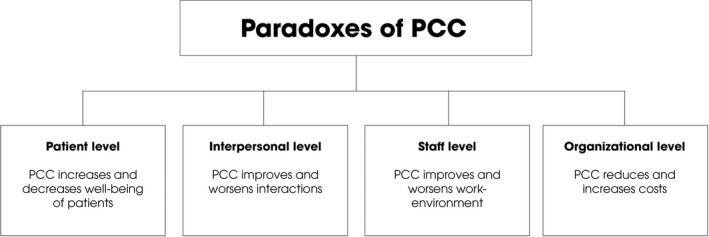
Four potential paradoxes of person‐centred care

Thus, *a first potential paradox* is that PCC might simultaneously increase and decrease patient well‐being. Second, an advantage of PCC appears to be improved mutual interactions while a disadvantage seems to favour some patients while ignoring others, which is unfair. Thus, *a second potential paradox* is that PCC at the same time tends to improve and worsen interactions. Third, PCC can contribute to an improved work environment while at the same time entailing risks of excluding staff's personhood and for compassion fatigue. Therefore, *a third potential paradox* is that PCC tends to both improve and worsen the work environment. Fourth, PCC appears to reduce costs, while at the same time it seems that it is relatively resource‐demanding; examples of costs are the consequences of falls and of compassion fatigue. Therefore, *a fourth potential paradox* is that PCC might both reduce and increase costs (Figure 1).

## 4 CONCLUSION

The PCC approach might be simultaneously underrated and overrated: it can be underrated by those who do not regard it as a powerful approach to creating a meaningful life for the patient, whereas it can be overrated by those who believe it is free from paradoxes and has only a positive impact on care. These two perspectives might come from a limited understanding of the relationships between health provider, patient(s), organizations, society and available resources. An awareness of these paradoxes might offer managers, staff and researchers insights when implementing, practicing and researching PCC. Future research may lead to a better understanding of the complexity of this phenomenon. It is likely that further research will find solutions to some of these paradoxes, while others might remain a dilemma.

## CONFLICT OF INTEREST

No conflict of interest has been declared by the authors.

## AUTHOR CONTRIBUTIONS

All authors made substantial contributions to conception and design, or acquisition of data, or analysis and interpretation of data; involved in drafting the manuscript or revising it critically for important intellectual content; given final approval of the version to be published and each author should have participated sufficiently in the work to take public responsibility for appropriate portions of the content; and agreed to be accountable for all aspects of the work in ensuring that questions related to the accuracy or integrity of any part of the work are appropriately investigated and resolved.
